# Second-Look Arthroscopy Shows Inferior Cartilage after Bone Marrow Stimulation Compared with Other Operative Techniques for Osteochondral Lesions of the Talus: A Systematic Review and Meta-Analysis

**DOI:** 10.1177/19476035241227332

**Published:** 2024-02-07

**Authors:** Jelmer T. Vreeken, Jari Dahmen, Tobias Stornebrink, Kaj S. Emanuel, Alex B. Walinga, Sjoerd A.S. Stufkens, Gino M.M.J. Kerkhoffs

**Affiliations:** 1 Department of Orthopedic Surgery and Sports Medicine, Amsterdam Movement Sciences, Amsterdam UMC, Location AMC, University of Amsterdam, Amsterdam, The Netherlands; 2 Academic Center for Evidence-based Sports Medicine (ACES), Amsterdam UMC, Amsterdam, The Netherlands; 3 Amsterdam Collaboration for Health & Safety in Sports (ACHSS), International Olympic Committee (IOC) Research Center, Amsterdam UMC, Amsterdam, The Netherlands

**Keywords:** ankle, talus, osteochondral lesion, second-look arthroscopy, cartilage

## Abstract

**Objective:**

To compare cartilage quality after different surgical interventions for osteochondral lesions of the talus (OLT), evaluated by second-look arthroscopy. Secondary aims were to report concomitant diagnoses, and to correlate cartilage quality with clinical and radiological outcomes. This review hypothesizes that the cartilage repair after bone marrow stimulation (BMS) is inferior to the other available treatment options.

**Methods:**

PROSPERO ID: CRD42022311489. Studies were retrieved through PubMed, EMBASE (Ovid), and Cochrane Library. Studies were included if they reported cartilage quality after second-look investigation after surgical treatment of OLT. The primary outcome measure was the cartilage quality success and failure rates (%) per surgical intervention group. Correlations between the cartilage quality and clinical or radiological outcomes were calculated.

**Results:**

Twenty-nine studies were included, comprising 586 ankles that had undergone second-look arthroscopy on average 16 months after initial surgery. The success rate for BMS was 57% (95% confidence interval [CI] = 48%-65%), for fixation (FIX) 86% (95% CI = 70%-94%), for osteo(chondral) transplantation (OCT) 91% (95% CI = 80%-96%), for cartilage implementation techniques (CITs) 80% (95% CI = 69%-88%), and for retrograde drilling 100% (95% CI = 66%-100%). The success rate of BMS was significantly lower than FIX, OCT, and CIT (*P* < 0.01). There were no significant differences between other treatment groups. A moderate positive significant correlation between the Magnetic Resonance Observation of Cartilage Repair Tissue (MOCART) score and the International Cartilage Repair Society score (ICRS) was found (ρ = 0.51, *P* < 0.001).

**Conclusions:**

Successful restoration of cartilage quality was found in the majority of surgically treated OLTs. However, BMS yields inferior cartilage quality compared with FIX, OCT, and CIT. *Study Design.* Systematic review and meta-analysis. *Level of evidence.* Level IV, systematic review and meta-analysis.

## Introduction

An osteochondral lesion of the talus (OLT) is defined as damage to the talar cartilage and its underlying subchondral bone,^1^ often caused by an ankle trauma.^2^ Compared with other articular cartilage, ankle cartilage is composed of identical cell types and matrix component.^3^ However, it differs in thickness, biomechanical, and biochemical properties, making it more susceptible for post-traumatic osteoarthritis.^3^ Hence, the fraction of post-traumatic etiology is the greatest in the ankle compared with other lower-limb joints.^4^ Since cartilage has limited intrinsic capacity to regenerate,^5^ it is a challenge to treat OLTs.

When conservative treatment fails (in 38% of the cases),^6^ a wide range of surgical options are available, such as bone marrow stimulation (BMS), retrograde drilling, internal fixation (FIX), osteo(chondral) transplantation (OCT), and chondrocyte implantation. However, up to now, there is no evidence for a superior treatment option for OLTs in terms of patient-reported outcomes.^6,7^ Clinical success rates of different treatments average between 75% and 85%, which means that 15%-25% of the patients has persistent symptoms after treatment for primary or secondary OLTs.^6^

A potential explanation for these persistent symptoms is that the cartilage may not be fully restored to hyaline-like cartilage, resulting in increased stress to the subchondral bone and also may predispose patients for osteoarthritis,^75^ which in turn is associated with physical and mental impairments.^8,9^ It is even thought that BMS leads—more so than other treatments—to cartilage regeneration of a nature other than hyaline-like cartilage. The confirmation of repair quality can be achieved through second-look arthroscopy, which allows a direct visual evaluation of the cartilage.^10^ Although it is not a standard procedure in clinical practice, several studies have used second-look arthroscopy as an outcome measure, providing an opportunity to compare the treatments’ capability to stimulate the cartilage regeneration of the OLTs.^11-13^ Insight in how successful the different treatments are in restoration of the cartilage may aid the clinician—and the patient alike—in their shared clinical decision making to determine the right treatment for each individual patient. An overview of the literature might support this clinical decision.

Therefore, this systematic review aims to assess the success and failure rates of cartilage repair of OLTs, as assessed during second-look arthroscopy, between different surgical techniques. We hypothesize that the cartilage repair after BMS is inferior to the other available treatment options. In addition, qualitative descriptions of the macroscopic appearance of cartilage and concomitant diagnoses will be evaluated. The secondary aim is to determine the relationship between cartilage quality and clinical or radiological outcomes.

## Methods

A systematic review of the literature was performed and was reported according to the methodology of the Preferred Reporting Items for Systematic Reviews and Meta-Analyses (PRISMA).^14^ The study was pre-registered at PROSPERO with ID: CRD42022311489.

### Search Strategy

The PubMed, EMBASE (Ovid), and Cochrane Library databases were searched from inception up to November 25, 2023 for potentially eligible articles. The following keywords were used: “osteochondritis dissecans,” “osteochrondrosis,” “osteochondrolysis,” “talar,” “osteochondral,” “lesion,” including synonyms and MeSH terms. The full search strategy is outlined in Appendix 1.

### Study Selection and Eligibility Criteria

Two independent reviewers (JV and JD) screened titles and abstracts using predefined criteria in Rayyan.^15^ Subsequently, articles were screened for full text and included when they met the inclusion criteria (**Table 1**). Discordant judgment in study inclusion was resolved by consensus discussion together with a third reviewer (KSE).

**Table 1.** Inclusion and Exclusion Criteria.

Inclusion	Exclusion
Clinical studies with a second-look arthroscopy after initial surgical treatment for OLTs of the talus	Second-look arthroscopy was performed only on patients with recurring or additional symptoms after the surgical intervention on the affected ankle
A cohort of five or more patients	Studies that did not present original data (e.g., reviews, technique papers, letters to the editor, editorial)
Written in English	Conference abstracts
	Animal or cadaveric studies
	Double publication including overlap of patients
	Full-text unavailable

OLT = osteochondral lesions of the talus.

### Methodological Quality Assessment

Methodological quality of the studies was evaluated using the validated Methodological Index for Non-Randomized Studies (MINORS) criteria^16^ by two independent reviewers.

### Data Extraction

The following study characteristics were retrieved: authors, year of publication, and study design. Also, the following patient characteristics were retrieved: gender, age, number of lesions, number of ankles, area, size, and location of the lesion, type of index surgical intervention, timing of second-look arthroscopy after index surgery, and methods for clinical and radiological assessment, and their corresponding outcomes.

### Outcome Measures

The primary outcome was the success and failure rates of cartilage repair as assessed during second-look arthroscopy and based on formal scoring systems.

Secondary outcomes were descriptions of the second-look macroscopic evaluation of the cartilage outside of scoring systems and the presence of concomitant diagnoses. Furthermore, correlations between cartilage repair scores and clinical or radiological outcomes were either collected or calculated.

### Treatment Classification

All included patient were assigned to treatment groups, using the grouping method as previously described by Steman *et al.*^17^

### Data Analysis

#### Primary outcome measure

We dichotomized all scoring systems in either “successful” or “failed” repair (**Table 2**). The International Cartilage Repair Society (ICRS) score^22^ was used as primary scoring system. Secondarily, the scoring systems by Nam *et al.*^18^ and Takao *et al.*^19^ were extracted if used as well. The overall dichotomized cartilage success and failure rates were pooled using a random effects model in R version 4.1.0 (R Core team, Vienna, Austria; metaprop function from the meta package^20^). The Clopper-Pearson interval was used to assess 95% confidence intervals (CIs). Differences in treatment success were compared between treatments using a moderator test for subgroup analysis with an α < 0.05 indicating statistical significance.^21^

**Table 2.** Description and Dichotomization of Grading Systems Included in Analyses.

	ICRS^22^	Takao *et al.*^19^	Nam *et al.*^18^
Successful	Normal (12) ^a^ Nearly normal (11-8) ^a^	0. Normal 1. Softening, or fibrillation 2. Fraying	3. Firm 2. Medium firm
Failure	Abnormal (7-4) ^a^ Severely abnormal (3-1) ^a^	3. Fragment detached but remains in crater 4. Fragment detached and loose	1. Soft and ballotable

ICRS = International Cartilage Repair Society score.^22^

a Corresponding score(s) to ICRS.

#### Secondary outcome measures

Qualitative descriptions of macroscopic cartilage appearance were categorized in “degree of integration to border zone” or “general macroscopic appearance,” “degree of defect repair,” and “other qualitative valuations.” For clarification purposes, subcategories were created (such as “Cartilage Appearance,” “Integration,” “Stableness,” “Firmness”). A numeric and percentage wise presentation of the ankles was given per description.

Concomitant diagnoses found during second-look arthroscopy were collected and pooled per treatment group.

Reported correlations between cartilage quality and clinical and radiological outcomes were collected. If the correlations were not described, but individual data were present, correlations were calculated using SPSS (Version 26. IBM Corp. Armonk, NY). A maximum window of 3 months between the mean second-look arthroscopy timing and either the mean clinical follow-up timing or the mean radiological follow-up timing per study was allowed for this analysis. In case of normally distributed data, Pearson’s correlation coefficients were calculated. Otherwise, Spearman’s rank-order correlation test was used.^23^

## Results

### Search Results

**Figure 1.**
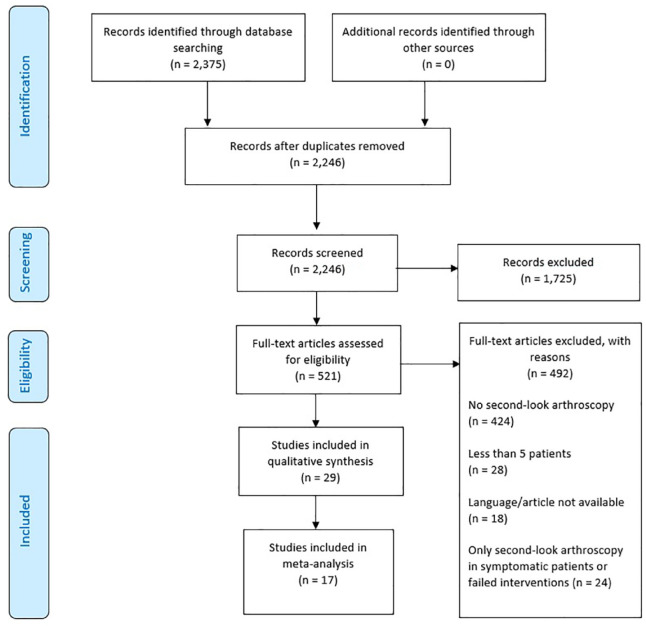
PRISMA 2009 flow diagram. PRISMA = Preferred Reporting Items for Systematic Reviews and Meta-Analyses.

After removal of duplicates, the literature search resulted in 2,246 articles. After screening titles and abstracts, 521 articles were included for full-text screening. A total of 29 articles were included for analysis (**Fig. 1**).

### Methodological Quality

The overall methodological quality of non-comparative studies ranged from 13% to 63% and for comparative studies from 46% to 79%. The MINORS criteria for comparative and non-comparative studies are shown in Appendices 2 and 3, respectively.

### Study Characteristics

The 29 studies reported a total of 872 lesions in 870 ankles in 862 patients. Of those 870 ankles, a total of 586 ankles (67%) underwent second-look arthroscopy and were included. The average age of the patients was 33 years (range among studies: 14-81 years) and 65% were men (range among studies: 31%-89%). The average preoperative lesion size was 181 mm^2^ (range among studies: 7-758 mm^2^). The use of preoperative radiological modality was reported in 26 studies (90%). The mean time of the second-look arthroscopy was 16 months (range among studies: 6-43 months) after the initial surgery (**Tables 3** and **4**). Of the 29 studies, four studies reported who performed the second-look arthroscopy (Appendix 4). One study reported that the second-look arthroscopy was assessed in random order by two independent observers.^2^ Another study reported that the second-look arthroscopy was reported by a different physician than the surgeon.^24^ The other two studies reported that the second-look arthroscopy was performed by the author(s).^11,25^

**Table 3.** Patient Characteristics.

Authors (Reference)	Study Design	Treatment Group	Patients (Ankles)	Number of Lesions	Males (%)	Ankles That Underwent SLA (%)	Age Mean Years* (Range)	Area Mean mm^2^* (Range)	Postoperative Radiology Used	Clinical Methods Used (Postoperative)	Evaluation Mean ± SD (Range)
Lee *et al.*^10^	PCS	BMS	19 (20)	20	15 (79%)	20 (100%)	38 (19-51)	90 (60-130)	Radiographs MRI	AOFAS	86.2 ± NR (78-97)
Takao *et al.*^11^	COHS	BMS	69 (69)	69	36 (52%)	68 (99%)	33 (16-57)	7 (5-10)**	NR	NA	NA
Yang and Lee^26^	PCS	BMS	25 (25)	25	16 (80%)	25 (100%)	NR (18-56)	NR	Radiographs MRI	AOFAS	90.0 ± NR (NR)
										VAS	2.0 ± NR (NR)
										FAOS	80.6 ± NR (NR)
										SF-36	75.0 ± NR (NR)
Giannini *et al.*^27^	PCS	BMS	48 (48)	48	27 (56%)	5 (10%)	29 (NR)	207	Radiographs MRI	AOFAS	88.9 ± 8.2 (72-100)
Lee *et al.*^28^	RCT	BMS	45 (45)	45	28 (62%)	8 (18%)	37 (NR)	103 (NR)	MRI	AOFAS	Group A: 91.23 ± 8.62 (NR) Group B: 86.91 ± 10.68 (NR)
										VAS	Group A: 17.25 ± 20.31 (NR)Group B: 19.37 ± 18.58 (NR)
										HSS	Group A 93.09 ± 13.64 (NR) Group B: 86.09 ± 13.36 (NR)
Takao *et al.*^29^	COHS	BMS	12 (12)	21	7 (33%)	21 (100%)	29 (14-49)	14 (10-17) **	MRI	NA	NA
		RD	9 (9)				28 (13-48)	13 (10-17) **			
Nakasa *et al.*^30^	RCOHS	FIX	17 (18)	18	10 (59%)	15 (83%)	20 (14-56)	330 (220-430)	MRI	NA	NA
Sawa *et al.*^31^	RCS	FIX	12 (12)	12	7 (58%)	7 (58%)	36 (14-70)	77 (30-110)	MRI CT	AOFAS	92 ± NR (90-100)
Choi *et al.*^32^	RCS	FIX	34 (34)	34	20 (59%)	21 (62%)	NR (11-29)	80 (NR)	MRI CT	Foot Function Index	NR
Kim *et al.*^12^	RCS	OCT	48 (52)	52	34 (71%)	52 (100%)	48 (21-67)	150 (52-294)	Radiographs	AOFAS	82.6 ± 7.8 (NR)
										VAS	3.3 ± 1.4 (NR)
										Tegner activity scale	3.9 ± 0.9 (NR)
										Satisfaction****	Excellent: 32 (62%) Good: 17 (33%) Fair: 3 (5%) Poor: 0 (0%)
Hu *et al.*^33^	RCS	OCT	17 (17)	17	16 (94%)	13 (76%)	37 (24-53)	14 (10-23) **	Radiographs MRI	NA	NA
Baltzer and Arnold^13^	PCS	OCT	43 (43)	43	30 (70%)	43 (100%)	31 (NR)	170 (NR-370)	Radiographs MRI	NA	NA
Zhu and Xu^34^	RCS	OCT	12 (12)	12	8 (67%)	5 (42%)	41 (34-60)	285 (150-500)	Radiographs MRI CT	NA	NA
Harada *et al.*^24^	PCS	OCT	10 (12)	12	8 (80%)	12 (100%)	46 (15-81)	103 (28-254)	Radiographs MRI	AOFAS	98.1 ± 2.8 (90-100)
Bai *et al.*^2^	RCS	OCT	19 (19)	19	14 (74%)	19 (100%)	35 (NR)	171 (85-758)	MRI CT	NR	NR
Shi *et al.*^25^	RCOHS	OCT	46 ( 46)	46	32 (70%)	32 (70%)	40 (NR)	NR	MRI CT	AOFAS	89.5 ± 8.7 (NR)
										VAS	1.5 ± 1.3 (NR)
										Tegner activity scale	4.8 ± 1 (NR)
Li *et al.*^35^	RCS	OCT	75 (75)	75	49 (63%)	8 (11%)	41 (19-60)	292 (150-NR)	MRI	AOFAS	82.8 ± 11.7 (NR)
										VAS	1.5 ± 1.3 (NR)
										SF-36	83.3 ± 8.5 (NR)
Yang *et al.*^36^	RCS	OCT	21 (21)	21	17 (81%)	11 (52%)	46 (NR)	5,039 (3,000-NR)***	MRI	AOFAS	90.9 ± 5.2 (NR)
										VAS	1.5 ± 0.9 (NR)
										FAOS	88.4 ± 6.3 (NR)
										AAS	4.6 ± 1.4 (NR)
Guo *et al.*^37^	RCS	OCT	26 (26)	26	20 (77%)	17 (65%)	42 (NR)	14 (NR)**	MRI CT	AOFAS	80.9 ± 10.0 (NR)
										VAS	4.0 ± 2.1 (NR)
										FFI	30.4 ± 18.4 (NR)
Nam *et al.*^18^	PCS	CIT	11 (11)	11	5 (45%)	10 (91%)	33 (21-47)	NR	MRI	NA	NA
Giannini *et al.*^38^	PCS	CIT	10 (10)	10	5 (50%)	10 (100%)	26 (16-49)	273 (NR)	Radiographs MRI	AOFAS	89.4 ± 14.5 (NR)
Giannini *et al.*^39^	PCS	CIT	8 (8)	8	4 (50%)	8 (100%)	28 (18-38)	310 (220-430)	Radiographs	AOFAS	90 ± NR (54-100)
Whittaker *et al.*^40^	PCS	CIT	10 (10)	10	7 (70%)	9 (90%)	42 (18-62)	NR	Radiographs	Mazur, Schwartz and Simon (modification)	73 ± NR (44-88)
Giannini *et al.*^41^	PCS	CIT	81 (81)	81	47 (58%)	19 (23%)	30 (NR)	195 (100-400)	Radiographs MRI	AOFAS	ACI: 87 ± NR (NR) BMDC: 89± 9 (NR)
López-Alcorocho *et al.*^42^	COHS	CIT	24 (24)	26	14 (58%)	24 (100%)	NR (18-55)	208	MRI	NA	NA
Ronga *et al.*^43^	PCS	CIT	6 (6)	6	5 (83%)	6 (86%)	29 (18-44)	340 (250-400)	MRI	NA	NA
Lee *et al.*^44^	PCS	CIT	38 (38)	38	33 (87%)	36 (95%)	35 (NR)	194 (NR)	Radiographs MRI	AOFAS	88 ± 8 (72-100)
										VAS	26 ± 24 (0-82)
										HSS	88 ± 12 (61-104)
Kwak *et al.*^45^	PCS	CIT	29 (29)	29	15 (52%)	25 (86%)	34 (16-54)	198 (NR)	Radiographs MRI	NA	NA
Lee *et al.*^46^	PCS	CIT	38 (38)	38	30 (79%)	38 (100%)	35 (16-55)	138 (NR)	MRI	NA	NA

SLA = second-look arthroscopy; PCS = prospective case series; BMS = bone marrow stimulation; MRI = magnetic resonance imaging; AOFAS = American Orthopedic Foot and Ankle Score; NR = not reported; COHS = prospective cohort study; NA = not applicable; VAS = Visual Analog Scale; FAOS = Foot and Ankle Outcome Score; SF-36 = 36-Item Short Form Survey; RCT = randomized controlled trial; HSS = Hannover Ankle Scoring System; RD = retrograde drilling; RCOHS = retrospective cohort study; FIX = fixation; RCS = retrospective case series; OCT = osteo(chondral) transplantation; CT = computed tomography; AAS = Ankle Activity Scale; FFI = Foot Function Index; CIT = Cartilage implantation techniques; ACI = autologous cartilage implementation; BMDC = bone marrow derived cells; OLT = osteochondral lesions of the talus.

* Only radiological/clinical timing and scoring system reported when used for analysis in the present study. **Median reported. ***Categorical scoring system reported.

* Rounded to integers, **Diameter of the OLT (mm), ***Volume of the OLT (mm^3^), ****Reported as “number of patients (percentage).”

**Table 4.** Study Characteristics.

Authors (Reference)	Treatment Group	Patients (Ankles)	Ankles Underwent SLA (%)	Follow-Up Timing After Index Surgery Mean Months (Range)			Second-Look Arthroscopy				
				SLA (Range)	Radiology (Range)	Clinical (Range)	Cartilage Scoring Systems	Successful Grades (Ankles)	Unsuccessful Grades (Ankles)	Success Rate (%)	ME Used
Lee *et al.*^10^	BMS	19 (20)	20 (100%)	12 (NR)	12 (NR)	12 (NR)	ICRS	I (3) II (9)	III (8) IV (0)	12/20 (60%)	Yes
							Ferkel and Cheng	A (2) B (3)	C (8) D (7)E (0) F (0)	5/20 (25%)	
Takao *et al.*^11^	BMS	69 (69)	68 (99%)	12 (NR)	NA	NA	System by Takao *et al.*^11^	0 (0) 1 (15) 2 (23)	3 (19) 4 (11)	38/68 (56%)	No
Yang and Lee^26^	BMS	25 (25)	25 (100%)	43 (26-97)	43 (26-97)	43 (26-97)	ICRS	I (3) II (13)	III (8) IV (1)	16/25 (64%)	No
							Ferkel and Cheng	A (5) B (6)	C (10) D (4) E (0) F (0)	11/25 (44%)	
Giannini *et al.*^27^	BMS	48 (48)	5 (10%)	12 (NR)	12 (NR)	12 (NR)	NR	NA	NA	NA	Yes
Lee *et al.*^28^	BMS	45 (45)	8 (18%)	24 (NR)	24 (NR)	24 (NR)	ICRS	I (7) II (1)	III (0) IV (0)	8/45 (100%)	No
Takao *et al.*^29^	BMS	12 (12)	21 (100%)	12 (NR)	12 (NR)	NA	ICRS	I (1) II (1)	III (7) IV (3)	2/12 (17%)	No
	RD	9 (9)						I (0) II (9)	III (0) IV (0)	9/9 (100%)	
Nakasa *et al.*^30^	FIX	17 (18)	15 (83%)	15 (12-18)	12 (NR)	NA	ICRS	I (4) II (8)	III (1) IV (0)	12/13 (92%)	Yes
Sawa *et al.*^31^	FIX	12 (12)	7 (58%)	12 (NR)	12 (NR)	NR	ICRS	I (4) II (3)	III (0) IV (0)	7/7 (100%)	Yes
Choi *et al.*^32^	FIX	34 (34)	21 (62%)	9 (6-45)	NA	9 (6-45)	ICRS	I (5) II (12)	III (3) IV (1)	17/21 (81%)	No
Kim *et al.*^12^	OCT	48 (52)	52 (100%)	13 (10-17)	NA	13 (10-17)	NR	NA	NA	NA	Yes
Hu *et al.*^33^	OCT	17 (17)	13 (76%)	12 (NR)	12 (NR)	NA	ICRS	I (1) II (11)	III (1) IV (0)	12/13 (92%)	Yes
Baltzer and Arnold^13^	OCT	43 (43)	43 (100%)	NR (6-9)	NA	NA	NR	NA	NA	NA	Yes
Zhu and Xu^34^	OCT	12 (12)	5 (42%)	NR (12-18)	NA	NA	ICRS	I (0) II (5)	III (0) IV (0)	5/5 (100%)	Yes
Harada *et al.*^24^	OCT	10 (12)	12 (100%)	12 (10-15)	NR	12 (NR)	ICRS	I (8) II (4)	III (0) IV (0)	12/12 (100%)	Yes
Bai *et al.*^2^	OCT	19 (19)	19 (100%)	24 (NR)	24 (NR)	NR	ICRS	I (9) II (9)	III (1) IV (0)	18/19 (95%)	No
Shi *et al.*^25^	OCT	46 (46)	32 (70%)	17 (NR)	NA	NR (24-60)	ICRS	NR	NR	NA	NR
Li *et al.*^35^	OCT	75 (75)	8 (11%)	NR	NA	NA	ICRS	I (2) II (4)	III (2) IV (0)	6/8 (75%)	Yes
Yang *et al.*^36^	OCT	21 (21)	11 (52%)	15 (NR)	NA	NR (36-NR)	ICRS	NR	NR	NA	NR
Guo *et al.*^37^	OCT	26 (26)	17 (65%)	12 (NR)	12 (NR)	12 (NR)	ICRS	I (12) II (4)	IV (1)	16/17 (94%)	Yes
Nam *et al.*^18^	CIT	11 (11)	10 (91%)	14 (9-24)	NA	NA	System by Nam *et al.*^18^	3 (3) 2 (4)	1 (3)	7/10 (70%)	Yes
Giannini *et al.*^38^	CIT	10 (10)	10 (100%)	15 (12-20)	NA	12 (NR)	NR	NA	NA	NA	Yes
Giannini *et al.*^39^	CIT	8 (8)	8 (100%)	12 (NR)	NA	12 (NR)	NR	NA	NA	NA	Yes
Whittaker *et al.*^40^	CIT	10 (10)	9 (90%)	13 (12-19)	NA	12 (NR)	NR	NA	NA	NA	Yes
Giannini *et al.*^41^	CIT	81 (81)	19 (23%)	12 (NR)	NA	12 (NR)	NR	NA	NA	NA	Yes
López-Alcorocho *et al.*^42^	CIT	24 (24)	24 (100%)	NR (12-NR)	NA	NA	NR	NA	NA	NA	Yes
Ronga *et al.*^43^	CIT	6 (6)	6 (100%)	6 (NR)	NA	NA	NR	NA	NA	NA	Yes
Lee *et al.*^44^	CIT	38 (38)	36 (95%)	12 (NR)	NA	12 (NR)	ICRS	I (5) II (22)	III (7) IV (0)	27/34 (79%)	Yes
							Mintz *et al.*^47^	0 (0) 1 (6)	2 (21) 4 (1) 3 (7) 5 (1)	6/36 (17%)	
							Six-point grading scale^44^	1 (7) 2 (7)3 (8)	4 (11) 5 (3)6 (0)	22/36 (61%)	
Kwak *et al.*^45^	CIT	29 (29)	25 (86%)	16 (NR)	NA	NA	ICRS	I (4) II (18)	III (2) IV (1)	22/25 (88%)	Yes
							System by Nam *et al.*^18^	3 (4) 2 (16)	1 (5)	20/25 (80%)	
Lee *et al.*^46^	CIT	38 (38)	38 (100%)	12 (NR)	12 (NR)	NA	Modified MOCART	NA	NA	NA	No

SLA = second-look arthroscopy; ME = macroscopic evaluation; BMS = bone marrow stimulation; NR = not reported; ICRS = International Cartilage Repair Society score; NA = not applicable since the difference in mean timing of the clinical/radiological assessment was > 3 months compared with the timing of SLA; RD = retrograde drilling; FIX = fixation; OCT = osteo(chondral) transplantation; CIT = cartilage implantation techniques; MOCART = Magnetic Resonance Observation of Cartilage Repair Tissue.

### Treatment Groups

The patients received the following surgical treatments: BMS (*n* = 138), retrograde drilling (*n* = 9), FIX (*n* = 43), OCT (*n* = 212), and cartilage implantation techniques (*n* = 185) (Appendix 4). The weighted mean timing of second-look arthroscopy after BMS, FIX, retrograde drilling, OCT, and cartilage implantation techniques, was 20, 11, 12, 14, and 13 months, respectively.

#### Success and failure rates

**Figure 2.**
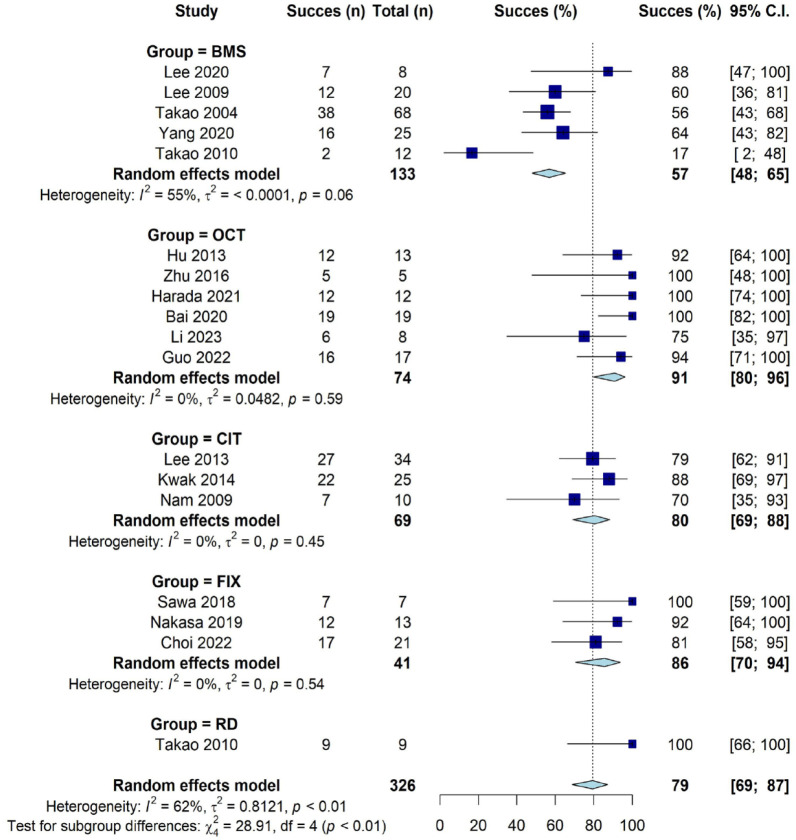
Forest plot of success rates of cartilage scoring systems by included studies. BMS = bone marrow stimulation; FIX = fixation; RD = retrograde drilling; OCT = osteo(chondral) transplantation; CIT = cartilage implantation techniques; ICRS = International Cartilage Repair Society score; CI = confidence interval.

Twenty studies used a cartilage scoring system (**Table 4**). The ICRS was used most frequent (17 studies; 59%). The pooled overall success rate was 79% (21% failure, 95% CI = 69-87; *I*^2^ = 62%; **Fig. 2**). The success rate of BMS (57%, 43% failure, **Fig. 2**) was significantly lower than FIX (86%, 14% failure; χ^2^ = 9.09.48, *P* < 0.01), OCT (91%, 9% failure; χ^2^ = 16.38, *P* < 0.01), and cartilage implementation techniques (CITs; 80%, 20% failure; χ^2^ = 9.88; *P* < 0.01). BMS and retrograde drilling (100%, no failure, from one study only) did not differ significantly. There were no significant differences in success rates between the other treatment groups (**Fig. 2**).

### Macroscopic Evaluation of the Repair Cartilage

Sixteen studies reported a qualitative description of macroscopic cartilage evaluation during second-look arthroscopy (**Table 5**). Regarding the macroscopic appearance of the cartilage for OCT and CITs, the described quality of the repair cartilage in all ankles were either “cartilage-like” or “hyaline-like.”

**Table 5.** Macroscopic Appearance.

Treatment	Category	Subcategory	Study	Macroscopic Evaluation (Description) of Repair Cartilage	Number of Patients (%)
BMS	Integration to border zone	Integration	Lee *et al.*^10^	Complete integration with the surrounding cartilage	6 (30%)
RD	NR	NR	NR	NR	NR
FIX	Other	Stableness	Nakasa *et al.*^30^	Stable^a^ lesions	15 (100%)
OCT	Integration to border zone	Integration	Kim *et al.*^12^	Uncovered area between plugs	14 (27%)
			Baltzer and Arnold^13^	Well integrated	43 (100%)
			Zhu and Xu^34^	Filled defect and consolidation of the surface with surrounding cartilage	5 (100%)
			Li *et al.*^35^	Continuous with the adjacent native articular cartilage	6 (75%)
		Level of graft	Zhu and Xu^34^	Leveled to adjacent cartilage	5 (100%)
	Macroscopic appearance	Cartilage appearance	Hu *et al.*^33^	“Cartilage-like”	13 (100%)
			Baltzer and Arnold^13^	Hyaline cartilage	43 (100%)
			Guo *et al.*^37^	Smooth	2 (12%)
				Fibrillated cartilage-like	14 (82%)
				Nearly bare bony appearance	1 (6%)
	Degree of defect repair	Surface	Kim *et al.*^12^	Uneven surface between two impacted plugs	10 (19%)
			Guo *et al.*^37^	Leveled to surrounding cartilage	2 (12%)
				Slightly higher than surrounding cartilage	14 (82%)
	Other macroscopic evaluations	Firmness	Hu *et al.*^33^	Softer at palpation than the surrounding cartilage	13 (100%)
			Zhu and Xu^34^	Firm	5 (100%)
		Stableness	Zhu and Xu^34^	Stable^a^	5 (100%)
CIT	Integration to border zone	Integration	López-Alcorocho *et al.*^42^	Graft was integrated	24 (100%)
			Whittaker *et al.*^40^	Complete marginal integration of the graft	8 (89%)
				Filled defect	9 (100%)
			Ronga *et al.*^43^	Defect was filled completely	6 (100%)
			Kwak *et al.*^45^	Good fill	25 (100%)
		Demarcation border	Nam *et al.*^18^	A line of demarcation between the graft and normal surrounding cartilage	10 (100%)
			Giannini *et al.*^39^	Slightly demarcated border	1 (13%)
		Level of graft	Whittaker *et al.*^40^	Raised graft compared with the surrounding cartilage	2 (22%)
	Macroscopic appearance	Cartilage appearance	Nam *et al.*^18^	Hyaline-like cartilage	10 (100%)
			Giannini *et al.*^38^	Hyaline-like cartilage	10 (100%)
			Ronga *et al.*^43^	Hyaline-like cartilage	6 (100%)
			López-Alcorocho *et al.*^42^	Normal or nearly normal	24 (100%)
	Degree of defect repair	Surface	Nam *et al.*^18^	Smooth surface	10 (100%)
			López-Alcorocho *et al.*^42^	Smooth surface	24 (100%)
			Giannini *et al.*^41^	Continuous cartilage layer	19 (100%)
			Giannini *et al.*^39^	Repaired cartilage covering the graft area	8 (100%)
			Kwak *et al.*^45^	Complete coverage of the defect and mild irregularity of the surface	25 (100%)
	Other macroscopic evaluations	Firmness	Nam *et al.*^18^	Softer at palpation than the surrounding cartilage	10 (100%)
			Giannini *et al.*^38^	Softer at palpation than the surrounding cartilage	10 (100%)
			Giannini *et al.*^39^	Softer at palpation than the surrounding cartilage	8 (100%)
			Whittaker *et al.*^40^	Softer at palpation than the surrounding cartilage	6 (67%)
		Stableness	Ronga *et al.*^43^	Stable^a^ at probing	6 (100%)
			López-Alcorocho *et al.*^42^	Stable^a^ at probing	24 (100%)
		Graft hypertrophy	Giannini *et al.*^39^	Moderate hypertrophy of the periosteal flap	2 (25%)
			Kwak *et al.*^45^	Graft hypertrophy	11 (38%)

SLA = second-look arthroscopy; BMS = bone marrow stimulation; RD = retrograde drilling; NR = not reported; FIX = fixation; OCT = osteo(chondral); CIT = cartilage implantation techniques.

a Stability was not explicitly defined.

### Concomitant Diagnoses

Eleven studies with a total of 213 patients (25%) reported concomitant diagnoses at second-look arthroscopy (**Table 6**). The most reported concomitant diagnosis was fibrous adhesion, which was found in 29% of the investigated OCT patients.

**Table 6.** Concomitant Diagnoses Per Treatment.

Treatment	Total Number of Patients	Weighed Mean Timing SLA	Concomitant Diagnose(s)	Number of Patients (%)
Bone marrow stimulation^10,29^	41	12	Fibrotic scar	7 (17%)
			Synovitis	2 (5%)
Fixation^30^	15	15	Synovitis	0 (0%)
Retrograde drilling^29^	21	12	Synovitis	0 (0%)
Osteo(chondral) transplantaion^12^	47	13	Fibrous adhesion	15 (29%)
			Synovitis	9 (19%)
Cartilage implantation techniques^18,38-41,45^	89	13	Periosteal hypertrophy	15 (17%)
			Synovitis	3 (4%)

SLA = second-look arthroscopy.

### Relation Between Second-Look Arthroscopy Cartilage Quality and Clinical Outcomes

Thirteen studies with a total of 258 patients (30%) reported clinical evaluation within 3 months of second-look arthroscopy (**Table 4**). The weighted mean timing of the follow-up of clinical assessment was 15 months after the index surgery. From these 13 studies, four had eligible data to be used in this review.

In a BMS cohort, Lee *et al.*^10^ reported higher mean American Orthopedic Foot and Ankle Society^48^ (AOFAS) scores in the successful repair group compared with the unsuccessful repair group, 88.7 (*n* = 12) versus 82.5 (*n* = 9), *P* = 0.005, at 12-month follow-up.

Yang and Lee^26^ reported that the mean Foot and Ankle Outcome Scores^49^ (FAOS) was higher in patients with successful cartilage repair, 86.8 (*n* = 16) versus 75.6 (*n* = 9), *P* < 0.05, at 43-month follow-up. Furthermore, Kim *et al.*^12^ found higher mean Visual Analog Scale^50^ (VAS) scores of pain in the presence of adhesion of the cartilage, 4.5 (*n* = 15) versus 3.6 (*n* = 37), *P* = 0.025, at 13-month follow-up. Also, a lower mean AOFAS score was found in the presence of adhesion, 78.4 (*n* = 15) versus 81.4 (*n* = 37), *P* = 0.005. In case of a graft with uncovered areas, a higher mean VAS, 4.8 (*n* = 14) versus 3.5 (*n* = 38), *P* = 0.002, and a lower mean AOFAS score, 72.1 (*n* = 14) versus 82.1 (*n* = 38), *P* = 0.001, were found.

Choi *et al.*^32^ reported that the ICRS and the Oswestry Arthroscopy score^51^ were negatively correlated with the Foot Function Index^52^ (Pearson’s correlation coefficient, r = −0.65, *P* = 0.001).

In a CITs cohort, Lee *et al.*^44^ reported a higher mean Hannover Scoring System^53^ (HSS) for the successful ICRS grades, 95 (*n* = 27) versus 85 (*n* = 9, *P* = 0.047). Neither the mean VAS nor mean AOFAS score was significantly different for successful versus unsuccessful grades.

### Relation Between Second-Look Arthroscopy Cartilage Quality and Radiology

Eleven studies with a total of 266 patients (31%) reported radiological evaluation within a 3-month window from second-look arthroscopy (**Table 4**). Seven of those studies, with a total of 164 patients (62%), reported a Magnetic Resonance Observation of Cartilage Repair Tissue^54^ (MOCART) score.

Yang and Lee,^26^ a BMS study, reported a median MOCART score of 68 (range = 30-95) corresponding to a median ICRS of 10 (range = 3-12) as well as a median Ferkel and Cheng^55^ grade of C (range = A-D). In addition, this study reported a discrepancy between magnetic resonance imaging (MRI) and second-look arthroscopy findings in six of the 25 cases (24%).

Analysis of individual patient data of three OCT studies^2,33,^37^^ showed a moderate positive significant correlation between the MOCART score and the ICRS (*n* = 42, ρ = 0.51, *P* < 0.001).

## Discussion

The most important finding of this review was that second-look arthroscopy showed successful restoration of cartilage quality in the majority of patients after different surgical treatments for OLTs. Successful cartilage repair was found in 80%-100% of the patients in most treatment groups. The success rate of BMS (57%) was markedly lower, which may suggest inferior repair capabilities.

It is known that BMS elicits a repair response by recruiting blood- and bone marrow-derived mesenchymal stem cells to injury site, where they stimulate the regeneration of the cartilage.^56^ A potential explanation for the fact that BMS yielded an inferior cartilage quality is that this surgical intervention does not preserve the native hyaline cartilage, but instead promotes the regeneration of cartilage. Regeneration often results in fibrocartilage,^57^ which has been shown to deteriorate over time.^58-61^ Furthermore, the weighted mean timing of second-look arthroscopy was 20 months for BMS compared with 12-14 months for the other treatments groups, which could have added to the inferior appearance of cartilage. It is not known whether the quality of the cartilage after BMS will decrease further after a longer follow-up period. Moreover, selection bias may be present due to the general consensus that BMS should be the primary option for relatively small defects.^62^ Another explanation may be that BMS has a potential negative effect on the quality of the subchondral bone plate, as shown by Reilingh *et al.*^63^ and Seow *et al.*^59^ An animal study on minipigs suggests that BMS in the knee leads to the irregularities of the subchondral bone plate as well as bone resorption after 6 months.^56^ It is known that irregularities may influence joint mechanics, uneven loading of the cartilage, and in severe cases to joint impingement.^56^

In quantitative analysis, we found high success rates for retrograde drilling (100%), FIX (86%), OCT (91%), and CITs (80%). These procedures all aimed to preserve the native cartilage,^44,64,65^ which may explain a satisfying macroscopic appearance upon second-look arthroscopic inspection. In the knee, similar numbers were reported when comparing autograft transplants with BMS after second-look arthroscopy (84% vs. 57%), which was confirmed by histological analysis of biopsies.^66^ For chondrocyte transplantation techniques, a significantly higher success rate (80%) was found than with BMS. A systematic review on BMS versus autologous chondrocyte transplantation in the knee cautiously concluded that the quality of the cartilage is higher after autologous chondrocyte transplantation.^76^ First-generation autologous chondrocyte transplantation studies found no significant differences in cartilage quality compared with BMS.^67^ Also, no difference in regeneration of tissue was found on MRI between the two treatments on talar defects.^68^ Reported qualitative analyses of the macroscopic appearance of the cartilage for OCT and CTIs was in line with the found high success rates in this review, as the quality of the repair cartilage in all ankles were described as either “cartilage-like” or “hyaline-like.”

The results of the cartilage quality of the retrograde drilling treatment are in line with pre-formed expectations, as the indication for this intervention is an intact layer of intra-articular hyaline cartilage and a degraded subchondral bone. The overlying cartilage will not be removed during the surgery, and therefore, no cartilaginous injury is expected to be uninjured directly after surgery.^69,70^ However, despite the perioperatively uninjured cartilage, second-look arthroscopy might still be relevant since an altered condition of the subchondral bone potentially affects the cartilage condition.

In general, macroscopic evaluation with second-look arthroscopy is limited to the surface of the repaired cartilage. Histological analysis is regarded as the gold standard in assessing cartilage quality, allowing assessment of the molecular structure of the cartilage, cellularity, integrity, and proteoglycan content throughout the entire thickness of the cartilage.^71^ However, a biopsy potentially damages the regenerated tissue, and represents only a fraction of the repaired area.^77^ Kok *et al.*^71^ reported that the macroscopic ICRS poorly correlates to the histological O’Driscoll score in a goat model. Furthermore, a low number of histological scoring systems for repaired osteoarthritic cartilage are actually currently validated.^78^ Second-look arthroscopy may be a less damaging alternative, and future studies should determine the correlation between second-look arthroscopy cartilage quality scoring systems, histological analyses, and clinical outcomes.

There were only four studies—two BMS studies, one OCT study, and one CIT study—that reported correlation between cartilage quality and clinical outcomes. The two BMS studies showed a moderate to strong positive correlation between the ICRS and the AOFAS and FAOS scores at 12 months postoperatively.^10,26^ Kim *et al.*^12^ showed a significant difference of OCT between the successful quality versus unsuccessful quality of the cartilage with their respective clinical scores, in contrast to Lee *et al.*,^44^ who found a mix of significant and non-significant differences between clinical scores and successful versus unsuccessful grades.

These findings imply that there might be a relationship between the cartilage quality and these clinical outcomes. Therefore, the low BMS cartilage success rates (57%)—as shown in this review—would suggest that also low clinical success rates would be achieved for BMS. However, Dahmen *et al.*^6^ reported clinical effectiveness—based on 52 studies—at medium-term follow-up (weighted means range from 26 to 52 months) for BMS (78%-86%). This indicates that good clinical outcomes at medium-term follow-up can be achieved despite an inferior success rate of the repair cartilage at a weighted mean of 16 months.

Eleven studies reported concomitant diagnoses in the ankle (ranging from 0% to 29%). In the OCT group, synovitis and adhesion were the most reported concomitant diagnoses during second-look arthroscopy. Kim *et al.*^12^ reported that significant differences were found between clinical outcomes (AOFAS, and VAS) and the presence or absence of adhesion. However, they also reported that no significant differences were found between clinical outcomes (AOFAS and VAS) and the presence nor absence of synovitis at the time of second-look arthroscopy.

This review found a significant moderate positive correlation between the MOCART and the ICRS score in 42 patients. Moreover, Lee *et al.*^72^ (*n* = 26 OLTs) compared MRI and arthroscopy 1 year after autologous chondrocyte implantation. They found that there was good reliability with respect to the categories “degree of defect repair,” “defect filling,” “cartilage repair,” and “synovitis.” However, one of the included studies reported a discrepancy between the MRI and second-look arthroscopy in 24% of the cases. Also, Lee *et al.*^72^ found poor to moderate reliability between MRI and arthroscopy for the categories “integration to border zone,” and “adhesion.” Therefore, it might be insufficient to use an MRI to assess the quality of the cartilage, and more suitable to perform second-look arthroscopy. However, a second-look arthroscopy is much more invasive than MRI. So, the physician must weigh the added value of a second-look arthroscopy compared with the MRI in a shared clinical decision with the patient.

This review has several limitations. First, selection bias might be present, as different populations were included for different treatments, and hence treatment groups lack comparability.^62^ Our main finding is that the quality of cartilage after BMS is inferior to other treatments. As BMS is usually the treatment that is implemented in relatively forgiving populations, with smaller size lesions and without history of prior surgery, it is not likely that selection bias is the reason for the poor outcome of this group. Second, The ICRS score was used as the main scoring system. Since the ICRS is validated for assessing the (repair) cartilage in the knee and is the most used system by the included studies in this review, the authors hypothesized that the scoring system was well applicable for scoring the ankle cartilage.^73^ However, high-quality validation studies for the ankle cartilage are necessary to confirm these results. Also, only 59% of the included studies reported this scoring system. Third, to be able to include all studies that systematically graded cartilage quality, other scoring systems (by Takao *et al.*^19^ and Nam *et al.*^18^) were used when the ICRS was not present, introducing potential heterogeneity in the analysis. Fourth, the mean timing of the second-look arthroscopy was 16 months. As a result, no conclusions can be drawn about the longer-term cartilage condition. Fifth, the treatment groups are relatively small. Hence, our statistical outcomes could be underpowered. Sixth, this review did not include histological examination in the analysis, as this fell outside the scope of our preregistered review. However, good correlation between histology and arthroscopy has been reported.^74^ Seventh, the overall quality of the studies ranged from 13% to 79%, introducing potential bias, mainly due to the study design of the included articles and the unbiased assessment of the study endpoints. Eighth, 31% of the studies only evaluated the cartilage qualitatively, making the description of the cartilage subjective, and hence less convenient to use their data for quantitative analysis. Finally, of the 29 included studies, only two studies^2,24^ reported that the assessed second-look arthroscopy was performed by a different observer than the surgeon who performed the initial surgery. In general, caution is advised with the interpretation of these outcomes. Since in clinical practice, clinical outcomes are leading in decision-making, failing to restore cartilage quality alone does not equal clinical failure. Therefore, further research is necessary to determine whether differences in cartilage quality lead to different clinical outcomes.

## Conclusion

Second-look arthroscopy after surgical treatment of OLTs of the talus shows successful cartilage scores in the majority (79%) of patients. Treatment with BMS showed a significantly lower success rate. Evaluation of cartilage quality with second-look arthroscopy correlated moderately with clinical outcome. Further research with appropriate study designs is necessary to determine the differences between treatment groups and the place of second-look arthroscopy in clinical practice.

## Supplemental Material

sj-docx-1-car-10.1177_19476035241227332 – Supplemental material for Second-Look Arthroscopy Shows Inferior Cartilage after Bone Marrow Stimulation Compared with Other Operative Techniques for Osteochondral Lesions of the Talus: A Systematic Review and Meta-Analysis Supplemental material, sj-docx-1-car-10.1177_19476035241227332 for Second-Look Arthroscopy Shows Inferior Cartilage after Bone Marrow Stimulation Compared with Other Operative Techniques for Osteochondral Lesions of the Talus: A Systematic Review and Meta-Analysis by Jelmer T. Vreeken, Jari Dahmen, Tobias Stornebrink, Kaj S. Emanuel, Alex B. Walinga, Sjoerd A.S. Stufkens and Gino M.M.J. Kerkhoffs in CARTILAGE

sj-docx-2-car-10.1177_19476035241227332 – Supplemental material for Second-Look Arthroscopy Shows Inferior Cartilage after Bone Marrow Stimulation Compared with Other Operative Techniques for Osteochondral Lesions of the Talus: A Systematic Review and Meta-Analysis Supplemental material, sj-docx-2-car-10.1177_19476035241227332 for Second-Look Arthroscopy Shows Inferior Cartilage after Bone Marrow Stimulation Compared with Other Operative Techniques for Osteochondral Lesions of the Talus: A Systematic Review and Meta-Analysis by Jelmer T. Vreeken, Jari Dahmen, Tobias Stornebrink, Kaj S. Emanuel, Alex B. Walinga, Sjoerd A.S. Stufkens and Gino M.M.J. Kerkhoffs in CARTILAGE

sj-docx-3-car-10.1177_19476035241227332 – Supplemental material for Second-Look Arthroscopy Shows Inferior Cartilage after Bone Marrow Stimulation Compared with Other Operative Techniques for Osteochondral Lesions of the Talus: A Systematic Review and Meta-Analysis Supplemental material, sj-docx-3-car-10.1177_19476035241227332 for Second-Look Arthroscopy Shows Inferior Cartilage after Bone Marrow Stimulation Compared with Other Operative Techniques for Osteochondral Lesions of the Talus: A Systematic Review and Meta-Analysis by Jelmer T. Vreeken, Jari Dahmen, Tobias Stornebrink, Kaj S. Emanuel, Alex B. Walinga, Sjoerd A.S. Stufkens and Gino M.M.J. Kerkhoffs in CARTILAGE

sj-docx-4-car-10.1177_19476035241227332 – Supplemental material for Second-Look Arthroscopy Shows Inferior Cartilage after Bone Marrow Stimulation Compared with Other Operative Techniques for Osteochondral Lesions of the Talus: A Systematic Review and Meta-Analysis Supplemental material, sj-docx-4-car-10.1177_19476035241227332 for Second-Look Arthroscopy Shows Inferior Cartilage after Bone Marrow Stimulation Compared with Other Operative Techniques for Osteochondral Lesions of the Talus: A Systematic Review and Meta-Analysis by Jelmer T. Vreeken, Jari Dahmen, Tobias Stornebrink, Kaj S. Emanuel, Alex B. Walinga, Sjoerd A.S. Stufkens and Gino M.M.J. Kerkhoffs in CARTILAGE

sj-docx-5-car-10.1177_19476035241227332 – Supplemental material for Second-Look Arthroscopy Shows Inferior Cartilage after Bone Marrow Stimulation Compared with Other Operative Techniques for Osteochondral Lesions of the Talus: A Systematic Review and Meta-Analysis Supplemental material, sj-docx-5-car-10.1177_19476035241227332 for Second-Look Arthroscopy Shows Inferior Cartilage after Bone Marrow Stimulation Compared with Other Operative Techniques for Osteochondral Lesions of the Talus: A Systematic Review and Meta-Analysis by Jelmer T. Vreeken, Jari Dahmen, Tobias Stornebrink, Kaj S. Emanuel, Alex B. Walinga, Sjoerd A.S. Stufkens and Gino M.M.J. Kerkhoffs in CARTILAGE

sj-docx-6-car-10.1177_19476035241227332 – Supplemental material for Second-Look Arthroscopy Shows Inferior Cartilage after Bone Marrow Stimulation Compared with Other Operative Techniques for Osteochondral Lesions of the Talus: A Systematic Review and Meta-Analysis Supplemental material, sj-docx-6-car-10.1177_19476035241227332 for Second-Look Arthroscopy Shows Inferior Cartilage after Bone Marrow Stimulation Compared with Other Operative Techniques for Osteochondral Lesions of the Talus: A Systematic Review and Meta-Analysis by Jelmer T. Vreeken, Jari Dahmen, Tobias Stornebrink, Kaj S. Emanuel, Alex B. Walinga, Sjoerd A.S. Stufkens and Gino M.M.J. Kerkhoffs in CARTILAGE

sj-docx-7-car-10.1177_19476035241227332 – Supplemental material for Second-Look Arthroscopy Shows Inferior Cartilage after Bone Marrow Stimulation Compared with Other Operative Techniques for Osteochondral Lesions of the Talus: A Systematic Review and Meta-Analysis Supplemental material, sj-docx-7-car-10.1177_19476035241227332 for Second-Look Arthroscopy Shows Inferior Cartilage after Bone Marrow Stimulation Compared with Other Operative Techniques for Osteochondral Lesions of the Talus: A Systematic Review and Meta-Analysis by Jelmer T. Vreeken, Jari Dahmen, Tobias Stornebrink, Kaj S. Emanuel, Alex B. Walinga, Sjoerd A.S. Stufkens and Gino M.M.J. Kerkhoffs in CARTILAGE

sj-docx-8-car-10.1177_19476035241227332 – Supplemental material for Second-Look Arthroscopy Shows Inferior Cartilage after Bone Marrow Stimulation Compared with Other Operative Techniques for Osteochondral Lesions of the Talus: A Systematic Review and Meta-Analysis Supplemental material, sj-docx-8-car-10.1177_19476035241227332 for Second-Look Arthroscopy Shows Inferior Cartilage after Bone Marrow Stimulation Compared with Other Operative Techniques for Osteochondral Lesions of the Talus: A Systematic Review and Meta-Analysis by Jelmer T. Vreeken, Jari Dahmen, Tobias Stornebrink, Kaj S. Emanuel, Alex B. Walinga, Sjoerd A.S. Stufkens and Gino M.M.J. Kerkhoffs in CARTILAGE
