# Supplementary material for: Second-Look Arthroscopy Shows Inferior Cartilage after Bone Marrow Stimulation Compared with Other Operative Techniques for Osteochondral Lesions of the Talus: A Systematic Review and Meta-Analysis
Source: Cartilage. 2024 Feb 7;17(1):36–51. doi: 10.1177/19476035241227332 (PMC11569557; doi:10.1177/19476035241227332)
Supplement: sj-docx-1-car-10.1177_19476035241227332 – Supplemental material for Second-Look Arthroscopy Shows Inferior Cartilage after Bone Marrow Stimulation Compared with Other Operative Techniques for Osteochondral Lesions of the Talus: A Systematic Review and Meta-Analysis [file sj-docx-1-car-10.1177_19476035241227332.docx]

**APPENDIX 1: Search strategy**

Search strategy until November 25 , 2023

| PUBMED | Search terms |
| --- | --- |
| #1 | "Osteochondritis Dissecans"[Mesh] |
| [#](https://www.ncbi.nlm.nih.gov/pubmed/advanced)2 | Osteochondritis dissecans[tiab] OR osteochondrosis dissecans[tiab] OR osteochondrolysis[tiab] OR OCD[tiab] OR OLT[tiab] |
| #3 | (osteochondral[tiab] OR chondral[tiab] OR transchondral[tiab] OR cartilage*[tiab]) AND (defect*[tiab] OR lesion*[tiab]) |
| #4 | #1 OR #2 OR #3 |
| #5 | "Talus"[Mesh] |
| #6 | talus[tiab] OR talar*[tiab] OR ankle[tiab] |
| #7 | #5 OR #6 |
| #8 | #4 AND #7 |
|  |  |
| EMBASE | Search terms |
| #1 | (osteochondritis dissecans/or (osteochondritis dissecans or osteochondrosis dissecans or osteochondrolysis or OCD or OLT).ti,ab,kw. or ((osteochondral or chondral or osteochondral or transchondral or cartilage*) adj3 (defect* or lesion*)).ti,ab,kw.) and (talus/ or (talus or talar* or ankle).ti,ab,kw.) |
|  |  |
| COCHRANE | Search terms |
| #1 | MeSH descriptor: [Osteochondritis Dissecans] explode all trees |
| #2 | osteochondritis dissecans or osteochondrosis dissecans or osteochondrolysis or OCD or OLT:ti,ab,kw (Word variations have been searched) |
| #3 | (osteochondral or chondral or transchondral or cartilage*) and (defect* or lesion*):ti,ab,kw (Word variations have been searched) |
| #4 | #1 or #2 or #3 |
| #5 | MeSH descriptor: [Talus] explode all trees |
| #6 | Talus or talar* or ankle*:ti,ab,kw (Word variations have been searched) |
| #7 | #5 or #6 |
| #8 | #4 and #7 |
