# Supplementary material for: Second-Look Arthroscopy Shows Inferior Cartilage after Bone Marrow Stimulation Compared with Other Operative Techniques for Osteochondral Lesions of the Talus: A Systematic Review and Meta-Analysis
Source: Cartilage. 2024 Feb 7;17(1):36–51. doi: 10.1177/19476035241227332 (PMC11569557; doi:10.1177/19476035241227332)
Supplement: sj-docx-2-car-10.1177_19476035241227332 – Supplemental material for Second-Look Arthroscopy Shows Inferior Cartilage after Bone Marrow Stimulation Compared with Other Operative Techniques for Osteochondral Lesions of the Talus: A Systematic Review and Meta-Analysis [file sj-docx-2-car-10.1177_19476035241227332.docx]

**APPENDIX 2: MINORS Criteria**^17^ **– Comparative Studies**

| **Criterium** | **Takao et al. (2010)^30^*** | **Takao et al. (2004)^12^** | **Lee et al. (2020)^29^** | **Shi et al (2022)^26^** | **Giannini et al. (2010)^42^** |
| --- | --- | --- | --- | --- | --- |
| Clearly stated aim | 2 | 2 | 2 | 2 | 2 |
| Inclusion of consecutive patients | 1 | 0 | 0 | 0 | 2 |
| Prospective data collection | 1 | 2 | 2 | 0 | 0 |
| Endpoints appropriate to aim of study | 1 | 1 | 2 | 2 | 1 |
| Unbiased assessment of study endpoint | 0 | 0 | 1 | 0 | 0 |
| Follow up period appropriate to aim of study | 1 | 1 | 2 | 2 | 1 |
| < 5% loss to follow-up | 0 | 0 | 0 | 0 | 0 |
| Prospective calculation sample size | 0 | 0 | 2 | 0 | 0 |
| Adequate control group | 2 | 2 | 2 | 2 | 2 |
| Contemporary groups | 0 | 0 | 2 | 1 | 1 |
| Baseline equivalence of groups | 1 | 2 | 2 | 2 | 0 |
| Adequate statistical analyses | 2 | 2 | 2 | 2 | 2 |
| **TOTAL % per study** | 46% | 50% | 79% | 54% | 46% |

MINORS-criteria points: 0 = Not reported, 1 = Reported but inadequate, 2 = Reported adequate

BMS = Bone marrow stimulation

CIT = Cartilage implantation techniques

RD = Retrograde drilling

*Study reported both BMS and RD treatment
