# Supplementary material for: Second-Look Arthroscopy Shows Inferior Cartilage after Bone Marrow Stimulation Compared with Other Operative Techniques for Osteochondral Lesions of the Talus: A Systematic Review and Meta-Analysis
Source: Cartilage. 2024 Feb 7;17(1):36–51. doi: 10.1177/19476035241227332 (PMC11569557; doi:10.1177/19476035241227332)
Supplement: sj-docx-3-car-10.1177_19476035241227332 – Supplemental material for Second-Look Arthroscopy Shows Inferior Cartilage after Bone Marrow Stimulation Compared with Other Operative Techniques for Osteochondral Lesions of the Talus: A Systematic Review and Meta-Analysis [file sj-docx-3-car-10.1177_19476035241227332.docx]

| **Study** | **Clearly stated aim** | **Inclusion of consecutive patients** | **Prospective data collection** | **Endpoints appropriate to aim of study** | **Unbiased assessment of study endpoint** | **Follow up period appropriate to aim of study** | **< 5% loss to follow up** | **Prospective calculation sample size** | **Total per study** |
| --- | --- | --- | --- | --- | --- | --- | --- | --- | --- |
| Lee et al. (2009)^11^ | 2 | 1 | 2 | 2 | 1 | 2 | 0 | 0 | 10/16 (63%) |
| Yang et al. (2020)^27^ | 2 | 1 | 1 | 2 | 2 | 1 | 0 | 0 | 9/16 (56%) |
| Giannini et al. (2009)^28^ | 0 | 0 | 2 | 2 | 0 | 2 | 2 | 0 | 8/16 (50%) |
| Choi et al (2022)^33^ | 2 | 1 | 0 | 2 | 0 | 2 | 0 | 0 | 7/16 (44%) |
| Nakasa et al. (2019)^31^ | 2 | 1 | 0 | 1 | 0 | 1 | 0 | 0 | 5/16 (31%) |
| Sawa et al. (2018)^32^ | 2 | 2 | 0 | 2 | 0 | 2 | 2 | 0 | 10/16 (63%) |
| Nam et al. (2009)^20^ | 2 | 0 | 2 | 1 | 0 | 1 | 0 | 0 | 6/16 (38%) |
| Kwak et al. (2014)^46^ | 1 | 2 | 2 | 2 | 1 | 1 | 0 | 0 | 9/16 (56%) |
| Giannini et al. (2009)^39^ | 2 | 1 | 1 | 1 | 0 | 1 | 0 | 0 | 6/16 (38%) |
| Giannini et al. (2001)^40^ | 1 | 1 | 1 | 1 | 0 | 1 | 0 | 0 | 5/16 (31%) |
| Whittaker et al. (2005)^41^ | 0 | 0 | 0 | 1 | 0 | 1 | 0 | 0 | 2/16 (13%) |
| López-Alcorocho et al. (2019)^43^ | 1 | 2 | 2 | 1 | 1 | 1 | 0 | 0 | 8/16 (50%) |
| Lee et al. (2013)^45^ | 2 | 1 | 1 | 1 | 0 | 1 | 0 | 0 | 6/16 (38%) |
| Lee et al. (2011)^47^ | 2 | 2 | 2 | 2 | 0 | 2 | 0 | 0 | 10/16 (63%) |
| Ronga et al. (2004)^44^ | 2 | 0 | 0 | 1 | 0 | 2 | 0 | 0 | 5/16 (31%) |
| Baltzer et al. (2005)^14^ | 0 | 1 | 2 | 1 | 0 | 1 | 0 | 0 | 5/16 (31%) |
| Hu  et al.  (2013)^2^ | 2 | 2 | 1 | 1 | 0 | 1 | 0 | 0 | 7/16 (44%) |
| Kim  et al. (2012)^13^ | 2 | 2 | 2 | 1 | 1 | 1 | 0 | 0 | 9/16 (56%) |
| Zhu & Xu (2016)^35^ | 2 | 1 | 0 | 1 | 0 | 1 | 0 | 0 | 5/16 (31%) |
| Harada et al. (2021)^25^ | 2 | 0 | 0 | 2 | 0 | 2 | 2 | 0 | 8/16 (50%) |
| Bai et al. (2020)^2^ | 2 | 0 | 0 | 2 | 0 | 2 | 0 | 0 | 6/16 (38%) |
| Li et al. (2023)^36^ | 2 | 0 | 0 | 2 | 1 | 2 | 2 | 0 | 9/16 (56%) |
| Yang et al. (2022)^37^ | 2 | 0 | 0 | 2 | 1 | 2 | 2 | 0 | 9/16 (56%) |
| Guo et al. (2022)^38^ | 2 | 2 | 0 | 2 | 1 | 2 | 1 | 0 | 10/16 (63%) |

**APPENDIX 3: MINORS Criteria**^60^ **– Non-Comparative Studies**

{Subtext ‘MINORS criteria points: 0 = Not reported, 1 = Reported but inadequate, 2 = Reported adequate

BMS = Bone marrow stimulation

FIX = internal fixation’}
