# Supplementary material for: Second-Look Arthroscopy Shows Inferior Cartilage after Bone Marrow Stimulation Compared with Other Operative Techniques for Osteochondral Lesions of the Talus: A Systematic Review and Meta-Analysis
Source: Cartilage. 2024 Feb 7;17(1):36–51. doi: 10.1177/19476035241227332 (PMC11569557; doi:10.1177/19476035241227332)
Supplement: sj-docx-4-car-10.1177_19476035241227332 – Supplemental material for Second-Look Arthroscopy Shows Inferior Cartilage after Bone Marrow Stimulation Compared with Other Operative Techniques for Osteochondral Lesions of the Talus: A Systematic Review and Meta-Analysis [file sj-docx-4-car-10.1177_19476035241227332.docx]

**APPENDIX 4: Reasons for second-look arthroscopy per study**

| **Authors (year publication)** | **Treatment group** | **Number ankles sla (% total ankles)** | **Basis for second-look arthroscopy** | **Additional action** | **Symptoms reported during SLA?** | **Reason symptoms** | **Number patients symptomatic (%)** | **Reported who performed SLA?** | **Who?** |
| --- | --- | --- | --- | --- | --- | --- | --- | --- | --- |
| Lee et al. (2009)^11^ | BMS | 20(100%) | Protocol | NR | NR | N.A. | N.A. | N.R. | N.A. |
| Takao et al. (2004)^12^ | BMS | 68(99%) | Protocol | NR | NR | N.A. | N.A. | Yes | “One of the authors” |
| Yang et al. (2020)^27^ | BMS | 25(100%) | Protocol | NR | NR | N.A. | N.A. | N.R. | N.A. |
| Giannini et al. (2009)^28^ | BMS | 5(10%) | Protocol | NR | Yes | NR | 2(40%) | N.R. | N.A. |
| Lee et al. (2020)^29^ | BMS | 8(18%) | Protocol | NR | NR | N.A. | N.A. | N.R. | N.A. |
| Takao et al. (2010)^30^ | BMS | 14(100%) | Protocol | NR | NR | N.A. | N.A. | N.R. | N.A. |
|  | RD | 11(100%) |  |  |  |  |  |  |  |
| Nakasa et al. (2019)^31^ | FIX | 15(83%) | Protocol | Hardware removal | NR | N.A. | N.A. | N.R. | N.A. |
| Sawa et al. (2018)^32^ | FIX | 7(58%) | Hardware removal | NR | No symptoms | N.A. | 0(0%) | N.R. | N.A. |
| Choi et al. (2022)^33^ | FIX | 21 (62%) | Hardware removal / Revision surgery | NR | NR | N.A. | N.A. | NR | N.A. |
| Kim et al. (2012)^13^ | OCT | 52 | Protocol | Hardware removal | NR | N.A. | N.A. | N.R. | N.A. |
| \|  \| Hu et al.  (2013)^34^ \| \| --- \| --- \| | OCT | 13 | Protocol | NR | NR | N.A. | N.A. | N.R. | N.A. |
| Baltzer et al. (2005)^14^ | OCT | 43(100%) | Protocol | Hardware removal | Yes | N.R. | 41(95%) | N.R. | N.A. |
| Zhu & Xu (2016)^35^ | OCT | 5(42%) | Hardware removal | NR | No symptoms | N.A. | 0(0%) | N.R. | N.A. |
| Harada et al. (2021)^25^ | OCT | 12(100%) | Protocol | Hardware removal | NR | N.A. | N.A. | Yes | Different physician than surgeon |
| Bai et al. (2020)^2^ | OCT | 19(100%) | Protocol | NR | NR | N.A. | N.A. | Yes | Assessed in random order by 2 independent observers |
| Shi et al 2022^26^ | OCT | 32 (70%) | Hardware removal | NR | NR | NR | NA | Yes | First and second author |
| Li et al. (2023)^36^ | OCT | 8 (11%) | Hardware removal | NR | NR | N.A. | N.A. | NR | N.A. |
| Yang et al. (2022)^37^ | OCT | 11 ( 52%) | Hardware removal or cultural reasons | NR | Yes | Hardware related | NR | NR | N.A. |
| Guo et al. (2022)^38^ | OCT | 17 (65%) | Hardware removal | NR | NR | N.A. | N.A. | NR | N.A. |
| Nam et al. (2009)^20^ | CIT | 10(91%) | Protocol | Hardware removal | NR | N.A. | N.A. | N.R. | N.A. |
| Giannini et al. (2009)^39^ | CIT | 10(100%) | Protocol | Hardware removal | NR | N.A. | N.A. | N.R. | N.A. |
| Giannini et al. (2001)^40^ | CIT | 8(100%) | Protocol | Checking fixation device | NR | N.A. | N.A. | N.R. | N.A. |
| Whittaker et al. (2005)^41^ | CIT | 9(90%) | Protocol | NR | NR | N.A. | N.A. | N.R. | N.A. |
| Giannini et al. (2010)^42^ | CIT | 19(23%) | Protocol | Hardware removal | NR | N.A. | N.A. | N.R. | N.A. |
| López-Alcorocho et al. (2019)^43^ | CIT | 24(100%) | Protocol | Hardware removal + biopsy | NR | N.A. | N.A. | N.R. | N.A. |
| Ronga et al. (2004)^44^ | CIT | 6(100%) | Protocol | Hardware removal | NR | N.A. | N.A. | N.R. | N.A. |
| Lee et al. (2013)^45^ | CIT | 36(95%) | Protocol | Hardware removal | NR | N.A. | N.A. | N.R. | N.A. |
| Kwak et al. (2014)^46^ | CIT | 25 | Protocol | Hardware removal | NR | N.A. | N.A. | N.R. | N.A. |
| Lee et al. (2011)^47^ | CIT | 38(100%) | Protocol | NR | NR | N.A. | N.A. | N.R. | N.A. |

BMS = Bone marrow stimulation, FIX = Fixation, RD = Retrograde drilling, OCT = Osteo(chondral) transplantation, CIT = Cartilage implantation techniques NR = Not Reported N.A. = Not Applicable.
