# Supplementary material for: Second-Look Arthroscopy Shows Inferior Cartilage after Bone Marrow Stimulation Compared with Other Operative Techniques for Osteochondral Lesions of the Talus: A Systematic Review and Meta-Analysis
Source: Cartilage. 2024 Feb 7;17(1):36–51. doi: 10.1177/19476035241227332 (PMC11569557; doi:10.1177/19476035241227332)
Supplement: sj-docx-5-car-10.1177_19476035241227332 – Supplemental material for Second-Look Arthroscopy Shows Inferior Cartilage after Bone Marrow Stimulation Compared with Other Operative Techniques for Osteochondral Lesions of the Talus: A Systematic Review and Meta-Analysis [file sj-docx-5-car-10.1177_19476035241227332.docx]

**APPENDIX 5: Included treatment groups (26 included studies) ***

| **Treatment** | **Number of surgical treatment options** |
| --- | --- |
| BMS ^11,12,27-30^  BMS without additional therapies  Matrix-assisted BMS | 6  4  2 |
| Fixation ^31,33^  Drilling + fixation  Autologous bone grafting with fixation of chondral fragments | 3  1  2 |
| Retrograde drilling^23^  Retrograde cancellous bone grafting | 1  1 |
| Osteo(chondral) transplantation ^2,13,14,25,26,32,34-38^  Autograft  Osteoperiosteal graft | 12  7  5 |
| Cartilage implantation techniques ^20,39-47^  Autologous chondrocyte implantation  Matrix induced autologous chondrocyte implantation | 10  9  1 |

*As there were studies that reported outcomes for multiple treatment options. The total number of treatment strategies is higher than the total number of included studies.

BMS = bone marrow stimulation
