# Supplementary material for: Second-Look Arthroscopy Shows Inferior Cartilage after Bone Marrow Stimulation Compared with Other Operative Techniques for Osteochondral Lesions of the Talus: A Systematic Review and Meta-Analysis
Source: Cartilage. 2024 Feb 7;17(1):36–51. doi: 10.1177/19476035241227332 (PMC11569557; doi:10.1177/19476035241227332)
Supplement: sj-docx-6-car-10.1177_19476035241227332 – Supplemental material for Second-Look Arthroscopy Shows Inferior Cartilage after Bone Marrow Stimulation Compared with Other Operative Techniques for Osteochondral Lesions of the Talus: A Systematic Review and Meta-Analysis [file sj-docx-6-car-10.1177_19476035241227332.docx]

**APPENDIX 6: Flow Chart of Included Treatment Groups**

**2,375** studies

**Exclusion criteria**

- No human subjects
- Not in English
- Less than 5 patients
- Etc. (Figure 1)

**29 studies** eligible to entry

*As there were studies that reported outcomes for multiple treatment options. The total number of treatment strategies is higher than the total number of included studies.

**5**

Operative groups

**12**

Osteo(chondral) Transplantation

**2**

Fixation of chondral fragments

**1**

Drilling and fixation

**2**

Matrix-assisted BMS

**4**

BMS without additional therapies

**6**

BMS

**1**

RD + cancellous bone graft

**1**

MACI

**9**

ACI

**5**

Osteo-periosteal graft

**7**

OAT

**10**

Cartilage

Implementation

Techniques

**1**

Retrograde drilling (RD)

**3**

Fixation

BMS = Bone marrow stimulation

FIX = internal fixation

CIT = Cartilage implantation techniques

OCT = Osteo(chondral) transplantation

OAT = Osteochondral autograft transfer

ACI = Autologous chondrocyte implantation

MACI = Matrix induced autologous chondrocyte implantation
